# Acute effects of resisted and assisted locomotor activation on sprint performance

**DOI:** 10.5114/biolsport.2022.108706

**Published:** 2022-01-25

**Authors:** Aleksander Matusiński, Artur Gołas, Adam Zajac, Adam Maszczyk

**Affiliations:** 1 Institute of Sport Sciences, The Jerzy Kukuczka Academy of Physical Education, Katowice, Poland; 2 Department of Exercise and Sport Performance, The Jerzy Kukuczka Academy of Physical Education in Katowice, Katowice, Poland

**Keywords:** PAP, Sprint performance, Athletes

## Abstract

Sprinting speed is one of the most significant motor abilities in team sport games and all sprint, hurdling and jumping events in athletics. Over the years numerous methods and devices have been developed to improve sprinting performance. One of the most effective methods of developing sprinting speed includes resisted sprinting with the use of different towing devices, parachutes, uphill running, and now intelligent drag technology. Resisted sprinting can be used for chronic changes in performance or for acute enhancement of running speed through locomotor post-activation. The other method of enhancing sprinting speed includes assisted sprinting in which the objective is to achieve supramaximal speed through high speed treadmill running, downhill sprinting, the use of elastic tubing or different towing mechanisms. The main objectives of this research were to determine the acute effects of resisted and assisted sprint activation on sprinting performance in male and female sprinters. Eleven, international and national level 200–400 m sprinters, 6 female and 5 male, participated in the study. The study protocol had a crossover design, with the activation protocol for both days consisting of either 3 × 30 m resisted sprints or 3 × 40 m assisted sprints. At baseline, and following the activation protocol, all athletes performed a 50 m maximum sprint, measured electronically with photocells from a crouched start. During particular trials, the SPRINT 1080 engine assisted measuring system was used with the load set individually to 10% BM. During the resisted and assisted PAP intervention the results of intragroup ANOVA revealed significant differences between resisted baseline results and resisted post activation results in the 10 m and 50 m test trials in men (respectively p = 0.002, η^2^ = 0.25; p = 0.001, η^2^ = 0.45), as well as in the group of female sprinters at these distances (10 m and 50 m) (respectively p = 0.002, η^2^ = 0.20; p = 0.001, η^2^ = 0.29). There were no statistically significant improvements in the 10 and 50 m test trials following assisted activation for both female and male sprinters. It was concluded that resisted sprint activation with a load of 10% BM enhances sprinting speed over 50 m in elite male and female sprinters.

## INTRODUCTION

Sprinting speed is an essential quality for numerous sport disciplines, yet it has a dominant effect on performance in athletics sprints, jumps and hurdle races. In the last several decades research has concentrated on biomechanics of running, and the effects of resistance – based training on sprint performance in general [1]. However most of the research on resistance training for speed development relates to team sports, with starting speed, acceleration and change of direction speed being the main study objectives [2, 3]. Studies specific to athletics, in particular acute and chronic interventions on sprinting performance are scarce [1, 4]. The goal of speed training is to increase the physical, metabolic, and neurological components essential for increasing sprinting speed [5]. Sprinting speed is a combination of stride length and stride frequency. To achieve high running velocity and improve sprinting speed, one or both of these variables must be improved. The most significant improvements in sprinting speed can be achieved by implementing training methods that stimulate both components of sprinting, stride length and stride frequency.

Sprinting speed can be enhanced by a variety of methods, which include resisted and assisted techniques [6]. Some specialists categorize these training methods into specific sprint training (free sprinting, resisted and assisted sprinting), and nonspecific training methods, which include strength, power and plyometric training, as well as combined or complex training methods, which are a mixture of specific and nonspecific methods [7]. Free sprint training, or sprint training without the use of any external equipment, forms the basis for most speed training programs, as it improves running technique, acceleration, top speed and speed endurance, depending on the choice of training variables [8]. Methods directed at improving stride frequency include high speed treadmill sprinting, elastic cord towing and downhill sprinting. On the other hand resistance – based methods enhance speed by increasing stride length. They include resisted sled towing, weighted vest running, uphill sprinting, strength training and plyometrics [1]. Recently, more advanced towing devices have been introduced to enhance sprinting speed and to monitor the training process. One of these technologically advanced training devices includes the SPRINT 1080 engine assisted measuring system which uses an intelligent drag technology to provide fully controlled resistance in the resisted and assisted phases of the sprinting movement [9]. Resisted sprint training allows to improve the athlete’s acceleration and maximum sprint speed as well. The main objective of resisted sprint training is to recruit more muscle fibers through greater neural activation, thus improving stride length [10, 11]. Towing can be used to induce acute neuromuscular changes by introducing several resisted sprints before free sprinting or it can be a part of a longer training program which promotes chronic adaptive changes resulting in increased stride length and improved acceleration mechanics [12]. Towing seems to have a greater impact on improving the acceleration phase, rather than maximum sprint speed, yet several key elements need to be considered for this method to be effective. Research has shown that insufficient resistance during towing may not produce a training stimulus, while excessive loading can alter sprint kinematics by increasing ground contact time, decreasing stride length and limiting hip extension [10, 13]. The most frequent recommendations for towing during sprint training include resistance between 10 and 13% body mass [14, 15], yet some research points to the fact that absolute strength of the lower limbs as well as the distance over which the towing takes place have a significant impact on the choice of the external loading. Weaker athlets, especially female sprinters may benefit more from resistance between 8 and 12% BM [16], while stronger male athlets can use loads of up to 40–45% BM at short towing distances (10–15 m) to improve acceleration [17].

Considering the above statements the main objectives of this research were to determine the acute effects of resisted and assisted sprint activation on sprinting performance in male and female sprinters.

## MATERIALS AND METHODS

### Participants

Eleven, international and national level 200–400 m sprinters, 6 female and 5 male, participated in the study. The research was carried out during a 7 day training camp at the beginning of the 2021 indoor season held at the Olympic Training Center. The training camp was preceded by several weeks of general conditioning and 6 weeks of specific explosive strength and sprint training. The average age, body mass and body height were 23.2 ± 5.4 years, 54.2 ± 6.1 kg, and 167.4 ± 5.3 cm for the female athletes, and 22.6 ± 2.8 years, 76.7 ± 4.3 kg, and 177.8 ± 4.3 cm respectively for male sprinters respectively. All of the athletes had at least 6 years of training and competition experience. The participants performed light technical exercises 24 hours prior to the intervention and testing to avoid fatigue. They were informed verbally and in writing about the procedures, possible risks and benefits of the study, and provided written consent before the commencement of the study. Moreover, they were asked to maintain their normal dietary and sleep habits throughout the study and not to use any supplements or stimulants for 24 h prior to testing. The study received the approval of the Bioethical Committee of the Academy of Physical Education in Katowice (10/2018), and was carried out according to the ethical standards of the Declaration of Helsinki, 2013.

### Procedures

During particular trials, the SPRINT 1080 engine assisted measuring system (1080 Motion AB, Stockholm, Sweden) was used for the precise selection of loads and variables, adapted to the diagnostics of sports training and performance [9]. The system uses changing intelligent drag technology to provide fully controlled resistance in the resisted and assisted phases of the movement. The device can record running time with an accuracy of 0.01 s and the average and peak values of such variables as force [N], power output [W] and velocity of a moving athlete [m/s]. The device has the option of changing setting of the resistance expressed in [kg] in all phases of the sprint. According to the data reported by the manufacturer, the system shows high repeatability and accuracy for measuring position (0.5%), velocity (≤ 0.5%), and force (≤ 4.8 N) [18].

The evaluations were carried out over 7 days, on Monday, Wednesday, Friday. Monday was dedicated to familiarization with the resisted and assisted sprint protocols, while on Tuesday and Thursday training sessions were composed of explosive strength exercises of high intensity and low volume to avoid fatigue before testing. The familiarization session was performed in order to minimize possible learning effects during the main tests. In order to eliminate the effects of weather conditions (wind, temperature etc.) on performance, the tests were performed on an indoor synthetic track. All activation, familiarization, and testing sessions were performed at the same time of the day, between 10:00 and 12:00 am. to avoid the influence of circadian rhythm on performance. On Monday, besides getting familiar with the experimental procedures the athletes were subjected to anthropometric measurements (height and weight). The participants used their track spikes during the activation protocol and during the speed evaluations. The research protocol was always preceded by a standardized, sprint specific warm-up (30–35 min) that was consistent with participants normal training habits. The study protocol had a crossover design, with the activation protocol for both days (Wednesday – Friday) consisting of either 3 × 30 m resisted sprints or 3 × 40 m assisted sprints, with 5 randomly chosen sprinters performing the resisted activation, while the remaining 6 were activated by assisted sprints. The distances differed in the resisted and assisted sprints to unify the time of the activation activity and metabolic cost, thus each activation sprint lasted approximately 5–6 s. Also previous testing of these athletes has shown that maximum velocity is reached between 33 and 37 m thus the distance for assisted sprints had to be extended beyond 30 m. The activation procedure was reversed on the second testing day. At baseline, and following the activation protocol, all athletes performed a 50 m maximum free sprint, measured electronically with photocells from a crouched start (Witty, Microgate, Bolzano, Italy). The resistance used during the activation protocol was set to 10% body mass, what was determined earlier empirically with a similar group of female and male sprinters [16]. The choice of this resistance was also in accordance with the recommendations of other authors, which suggest values of 10–13% BM for maximal enhancement of sprinting speed [14, 15]. The assisted sprints used the same load of 10% BM, yet the athletes were pulled by the 1080 device, what corresponded to towing speeds of 110–112% unassisted values. The intensity of the assisted sprints was also in line with previous research and recommendations of other authors [19]. Five minutes after the baseline 50 m sprint, a set of 3 × 30 m resisted or 3 × 40 m assisted sprints were performed with 5–6 min rest intervals in between to ensure full recovery. Considering the maximal or supramaximal intensity of the activation exercise, the authors chose a 8 min recovery to optimize potentiation before the second 50 m test trial. The choice of such a rest interval was based on previous research and recommendations of several authors [3, 20, 21].

The 50 m test trials evaluated starting speed, acceleration and maximum velocity, and started from a crouched start, without external resistance. After receiving a verbal signal, the participant started at will. After an additional 5 min rest interval, one of the 2 activation protocols was performed in random order. The activation and testing took place on Wednesday and Friday at the same time of day, under identical conditions, with Thursday being a day of recovery and brief explosive strength training.

The main hypothesis of the study was that acute activation with resisted and assisted sprints enhances sprint performance in female and male sprinters, yet through different mechanisms.

### Statistical analysis

All statistical analyses were performed using STATISTICA (Stat Soft, Inc. 2021, version 13), while activation protocol and PAP results were expressed as mean ± SD. The Shapiro-Wilk, Levene and Mauchly´s tests were used in order to verify the normality, homogeneity and sphericity of the sample’s data variances, respectively.

The multivariate ANOVA was used with significance set at p<0.05, to determine differences between resisted and assisted exercises in male and female athletes for activation protocol time, peak power, force and velocity variables. When appropriate, a Tukey post hoc test was used to compare selected data, and the effect of each test was calculated to determine the significance of the results. The same ANOVA analysis protocol was used to determine differences between baseline and post activation PAP results for resisted and assisted activation in female and male athletes. Effect sizes (Cohen’s d) were reported where appropriate. According to Hopkins guidelines, the effect size (eta-squared; η^2^) was established as follows: 0.01 – small, 0.06 – medium, and 0.14 – large [22].

## RESULTS

The intragroup ANOVA analysis between activation protocol variables revealed significant differences between resisted 30 m and assisted 40 m sprints in time and velocity in the group of female athletes (respectively p = 0.016, η^2^ = 0.07; p = 0.006, η^2^ = 0.14), and for velocity only in male sprinters (p = 0.001, η^2^ = 0.27). There were no statistically significant intragroup differences between men’s resisted 30 m and assisted 40 m in in time, peak power and force, as well as in women’s peak power and force (Table 1).

**TABLE 1:** The intragroup and intergroup ANOVA analysis between activation protocol variables in female and male sprinters.

Activation Protocol					
**Group**	**Exercise**	**Time (s)**	**p (re. vs ass.)**	**Peak Power (W)**	**p (re. vs ass.)**
MEN	Resisted 30 m (10% BM)	4.94 ± 0.12 **	0.502	973 ± 29**	0.294
	Assisted 40 m (10% BM)	4.98 ± 0.14 **		1007 ± 35**	
WOMEN	Resisted 30 m (10% BM)	5.35 ± 0.11	0.016*	473 ± 33	0.377
	Assisted 40 m (10% BM)	5.23 ± 0.09		489 ± 45	

**Group**	**Exercise**	**Force (N)**	**p (re. vs ass.)**	**Velocity (m/s)**	**p (re. vs ass.)**
MEN	Resisted 30 m (10% BM)	112 ± 21**	0.289	8.33 ± 1.12	0.001*
	Assisted 40 m (10% BM)	123 ± 24**		11.9 ± 1.21**	
WOMEN	Resisted 30 m (10% BM)	64 ± 25	0.619	7.88 ± 1.15	0.006*
	Assisted 40 m (10% BM)	58 ± 28		10.26 ± 1.43	

Note:

* significant intragroup differences between resisted and assisted;

** significant intergroup differences between groups of male and female sprinters.

The intergroup ANOVA analysis between activation protocol variables revealed significant differences between resisted 30 m results in time, peak power and force (respectively p = 0.001, η^2^ = 0.25; p = 0.001, η^2^ = 0.27; p = 0.001, η^2^ = 0.20), as well as between assisted 40 m results in time, power, force and velocity (respectively p = 0.001, η^2^ = 0.23; p = 0.001, η^2^ = 0.28; p = 0.001, η^2^ = 0.35 and p = 0.002, η^2^ = 0.18). There was no statistically significant intergroup differences between resisted 30 m results in velocity (Table 1).

During the resisted and assisted PAP intervention the results of intragroup ANOVA revealed significant differences between resisted baseline results and resisted post activation results in the 10 m and 50 m test trials in men (respectively p = 0.002, η^2^ = 0.25; p = 0.001, η^2^ = 0.45), as well as in the group of female sprinters at these distances (10 m and 50 m) (respectively p = 0.002, η^2^ = 0.20; p = 0.001, η^2^ = 0.29). There were no statistically significant improvements in the 10 and 50 m test trials following assisted activation for both female and male sprinters (Table 2).

**TABLE 2:** The intragroup and intergroup ANOVA analysis between baseline and post activation PAP results in female and male sprinters.

PAP Results						
	Resisted			Assisted		
Distance	Baseline	Post Activation	p (bas. vs post-activ.)	Baseline	Post Activation	p (bas. vs post-activ.)
**MEN**						
10 m (s)	1.77 ± 0.05**	1.66 ± 0.04**	0.002	1.73 ± 0.05**	1.72 ± 0.03**	0.613
50 m (s)	6.01 ± 0.09**	5.86 ± 0.07**	**0.001**	5.97 ± 0.11**	5.92 ± 0.09**	0.345

**WOMEN**						
10 m (s)	1.92 ± 0.06	1.87 ± 0.06	0.002	1.90 ± 0.05	1.92 ± 0.05	0.663
50 m (s)	6.63 ± 0.11	6.58 ± 0.09	0.001	6.60 ± 0.12	6.62 ± 0.11	0.654

Note:

** significant intergroup differences between male and female sprinters.

The intergroup ANOVA analysis revealed significant differences between men’s and women’s resisted baseline results and resisted post activation results in the 10 m and 50 m test trials (Table 2).

## DISCUSSION

Sprinting speed is of great interest to scientists, athletes and coaches in numerous sport disciplines, as this motor ability plays a key role in performance. Sprinting performance has captured the attention of spectators since the ancient Olympics [23], yet more attention has been paid to the analysis of sprinting mechanics and physiology since the modern Olympic games and the development of numerous team sport games in which running speed has a significant effect on sports performance. The end of the XX century and especially the XXI century has brought an enormous advancement in technology, that has been applied both to training and scientific research which includes physiological changes [24, 25], kinematic studies [26, 27] and the introduction of training devices used for assisted and resisted sprint training [14, 16, 28]. Considering methodological issues team sport athletes are easier to study because of the greater number of homogenous participants, as in American football, rugby or soccer, which can be divided into intervention and control groups. This is why there is significantly less studies on elite sprinters of both genders than data on competitive team sport players [1, 16]. Additionally there is also a greater abundance of acute studies with resistance interventions on speed improvements, while most of the well-controlled chronic research has been performed on team sport athletes. Thus the novelty of our research includes the choice of the study participants, which included elite national sprinters of both genders. All of them trained for at least 6 years and competed at the national and international level. Additionally we used 2 familiarization sessions to acquaint the athletes with the apparatus used for resisted and assisted sprinting. Previously all the athlets used sled towing during resistance sprinting and elastic tubing in assisted training, yet none of them have used the SPRINT 1080 engine assisted measuring system. This was another novelty of the research as this device uses changing intelligent drag technology to provide fully controlled resistance in the resisted and assisted phases of the movement. By recording time of the activation with an accuracy of 0.01 s, as well as other variables such as peak force [N], peak power output [W] and velocity of the moving athlete [m/s] we were able to compare the two activation protocols. The last yet significant aspect of the research was the empirically chosen rest intervals between the activation activity and the free sprint, which was based on previous studies on PAP in sprints and other resistance exercise protocols [9, 16, 20, 29, 30].

The objective of the activation protocols was to reach acute enhancement of sprinting speed, yet through different mechanisms in case of the resisted and assisted exercises. The chosen distance for the assisted activation was increased to 40 m to unify the time of the effort and possible energy cost. Additionally the goal of the assisted sprints was to reach top speed at the 30–35 m mark due to the much greater acceleration possible through the pulling action of the Sprint 1080 device. Under natural conditions top speed is most often reached at 45–50 m in female athletes and at 55–60 m in male sprinters [27]. It must be indicated that the time of the activation protocols (resisted & assisted) was very similar in case of male and female sprinters, while peak power and peak force also did not differ significantly between the two types of activation. Naturally due to the greater body mass and much higher strength potential of the male sprinters their peak power and peak force generated during the activation protocols were nearly twice as high as those of female athletes. The significant difference between the two activation protocols occurred in case of running velocity, which was 3.57 m/s greater in male sprinters for the assisted sprints and 2.38 m/s higher for assisted sprints in case of female athletes.

The results of our experiment with male and female sprinters, showed positive acute effects of the intervention on sprint performance, evaluated by free sprinting from a crouch start over 50 m with photo-timing. However, the effects were only visible following activation with resisted sprints. These effects were observed for both male and female sprinters, yet the margin of improvement was greater for male athletes. This could be explained by the greater strength potential of the male sprinters [3, 14]. Resisted sprint activation over 30 m with the resistance set at 10% BM resulted in improved starting speed evaluated by the time over 10 m, as well as maximum speed measured at a distance of 50 m. This phenomenon can be explained by the previous contractile history termed post activation potentiation (PAP) [9, 16, 17]. It seems that the appropriate PAP variables which included empirically verified load and rest intervals, as well as the biomechanical similarity of the activation and test trial exercise, allowed for increased neural drive and increased stiffness of the muscle-tendon complex involved in sprinting [29]. As mentioned before, previous research has shown that greater PAP effects are observed for stronger athletes, and this was confirmed in case of the male sprinters [20, 21]. The assisted activation protocol did not enhance performance, neither for starting speed nor for maximum sprinting performance, despite 2 familiarization sessions. It seems that the athletes were more accustomed to resisted towing, which stimulated specific muscles without disrupting sprinting technique. On the other hand the athletes were less familiar with overspeed training, and perhaps the assisted force was too high, reaching velocities in the 110–112% range of max free sprinting speed. Excessive velocity associated with the assisted activation may have caused overstriding and a loss of coordination, what did not allow for acute enhancement of sprint performance [31, 32]. Overspeed training through assisted sprinting may require significant neuromuscular adaptations related to increased efficiency of motor unit synchronization, through repeated training sessions over a longer period of time [33]. Such interventions should relate to chronic adaptive changes planed over a 4–6 week meso-cycle.

Despite several novel aspects, the study has several limitations. First of all the number of participants was rather small, yet it was difficult to invite more elite sprinters to the project. Second of all, the sprinters were much more familiar with resisted sprint training in the form of towing, while few had experience with assisted sprint training. There is a insufficiency of research in the field of overspeed training, especially related to the intensity of the pulling force. Our research lacked kinematic evaluations, which would have helped in evaluating the effects of resisted and assisted activation on stride length and stride frequency.

## CONCLUSIONS

Resistance sprint training with the use of the Sprint 1080 drag technology device with a load of 10% BM is effective in acute enhancement of starting and maximum sprinting speed. On the other hand assisted activation with the same load does not enhance sprinting performance. It seems that the pulling force may be to high generating extreme velocities which cause over-striding and changes in sprinting technique. Assisted sprint training requires a longer familiarization period and most likely pulling forces which allow to reach velocities of 105–107% of maximal free sprinting speed.

## Author contributions

All authors contributed in a complementary way to the design and implementation of the research, to the analysis of the results and to the writing of the manuscript.

## Conflict of interests

The authors declared no conflict of interests regarding the publication of this manuscript.
